# Inotuzumab ozogamicin is effective in relapsed/refractory extramedullary B acute lymphoblastic leukemia

**DOI:** 10.1186/s12885-018-5026-x

**Published:** 2018-11-15

**Authors:** Luca Bertamini, Jacopo Nanni, Giovanni Marconi, Mariachiara Abbenante, Valentina Robustelli, Francesco Bacci, Antonella Matti, Stefania Paolini, Chiara Sartor, Silvia Lo Monaco, Maria Chiara Fontana, Stefano De Polo, Michele Cavo, Antonio Curti, Giovanni Martinelli, Cristina Papayannidis

**Affiliations:** 1 DIMES (Department of Experimental, Diagnostic, and Specialty Medicine), Institute of Hematology “L. and A. Seragnoli”, University of Bologna, Via Massarenti 9, 40138 Bologna, Italy; 2 DIMES (Department of Experimental, Diagnostic, and Specialty Medicine), Nuclear Medicine Division, University of Bologna, Bologna, Italy; 3 Hematology Unit, Istituto Scientifico Romagnolo per lo Studio e la Cura dei Tumori IRCCS Meldola, Meldola, Italy

**Keywords:** Inotuzumab ozogamicin, Extramedullary, Acute lymphoblastic leukemia, PET-CT scan

## Abstract

**Background:**

Extramedullary involvement of B-cell Acute Lymphoblastic Leukemia (EM-ALL) is a rare occurrence, characterized by dismal outcome and the absence of a defined and shared therapeutic approach. In the landscape of innovative compounds, inotuzumab ozogamicin (IO) is a promising drug, whose mechanism of action relies on the killing of CD22 positive leukemic cells, through the delivery, after cell binding, of a molecule of calicheamicin.

**Case presentation:**

We report two cases of CD22 positive relapsed EM-ALL treated with IO, obtained as compassionate use. Case 1, a 66 years old woman, affected by Philadelphia (Ph) negative B-ALL, relapsed with extramedullary involvement after 6 standard chemotherapy courses, who reached a complete metabolic response with IO treatment. Case 2, a 67 years old man with Ph positive B-ALL, initially treated with ponatinib, a third generation tyrosine-kinase inhibitor (TKI), obtaining a prolonged deep molecular remission. Nevertheless, for skin relapse during TKI treatment, the patient received local radiotherapy and, shortly after, standard chemotherapy, as multiple abdominal sites of relapse were detected too, with no response. The patient then received IO, obtained as compassionate use, with a good metabolic response.

**Conclusions:**

These two cases suggest a possible key role of IO in the setting of advanced CD22 positive ALL, and underline its potential activity also in patients with EM involvement, relapsed after or refractory to conventional chemotherapy. Despite the well known hepatotoxic effect of the compound (Sinusoid Occlusive Syndrome), neither of them had such adverse event, moreover the second patient safely underwent allogeneic bone marrow transplantation.

## Background

Salvage therapy of relapsed/refractory (R/R) B-cell Acute Lymphoblastic Leukemia (ALL) remains an unsolved issue [1]. Aside standard chemotherapy, which is the best treatment choice between blinatumomab, CAR-T cells and IO, that currently represent the most promising therapeutic options? [2] To the best of our knowledge, there are no shared guidelines on the management of R/R B-ALL, even more considering the particular setting of EM disease. On one hand, despite the impressive activity demonstrated in the setting of Minimal Residual Disease (MRD) positive ALL patients, blinatumomab seems to be less effective in the context of EM involvement [3]. On the other hand, the recent clinical trials assessing the role of IO in R/R patients did not specifically focus on EM disease. Therefore, the investigation of the role of IO in patients with such a poor prognosis is strongly required.

## Case presentation

### Case1

A 66 years old woman, presenting increasing asthenia, revealed at peripheral blood count a severe anemia (Hb 7.3 g/dL), a reduced white blood cell (WBC) count (2400/μL) with severe neutropenia (neutrophils 600/μL), and a normal platelet count (PLT 168.000/μL). The diagnostic work-up showed a B-ALL, with normal karyotype, negative for BCR-ABL rearrangement and with immature B-cell origin (CD19+, CD22+, SMIg+, TdT+, CD20-). The CT-scan performed at diagnosis revealed a solid-lesion (7.0 cm width) at the right kidney’s inferior pole, that turned out to be a clear cell carcinoma (surgically removed later). The patient received 6 courses of chemotherapy according to BFM schedule, following local Institutional guidelines, including monthly intrathecal central nervous system (CNS) prophylaxis. After the first chemotherapy cycle, she reached a morphologic complete remission (CR) with MRD negativity, evaluated by analysis of clonal rearrangement of IgH gene study (according to Biomed EuroMRD Protocol [4]). Such a deep response was confirmed and maintained during all the six courses of chemotherapy.

**Fig. 1:**
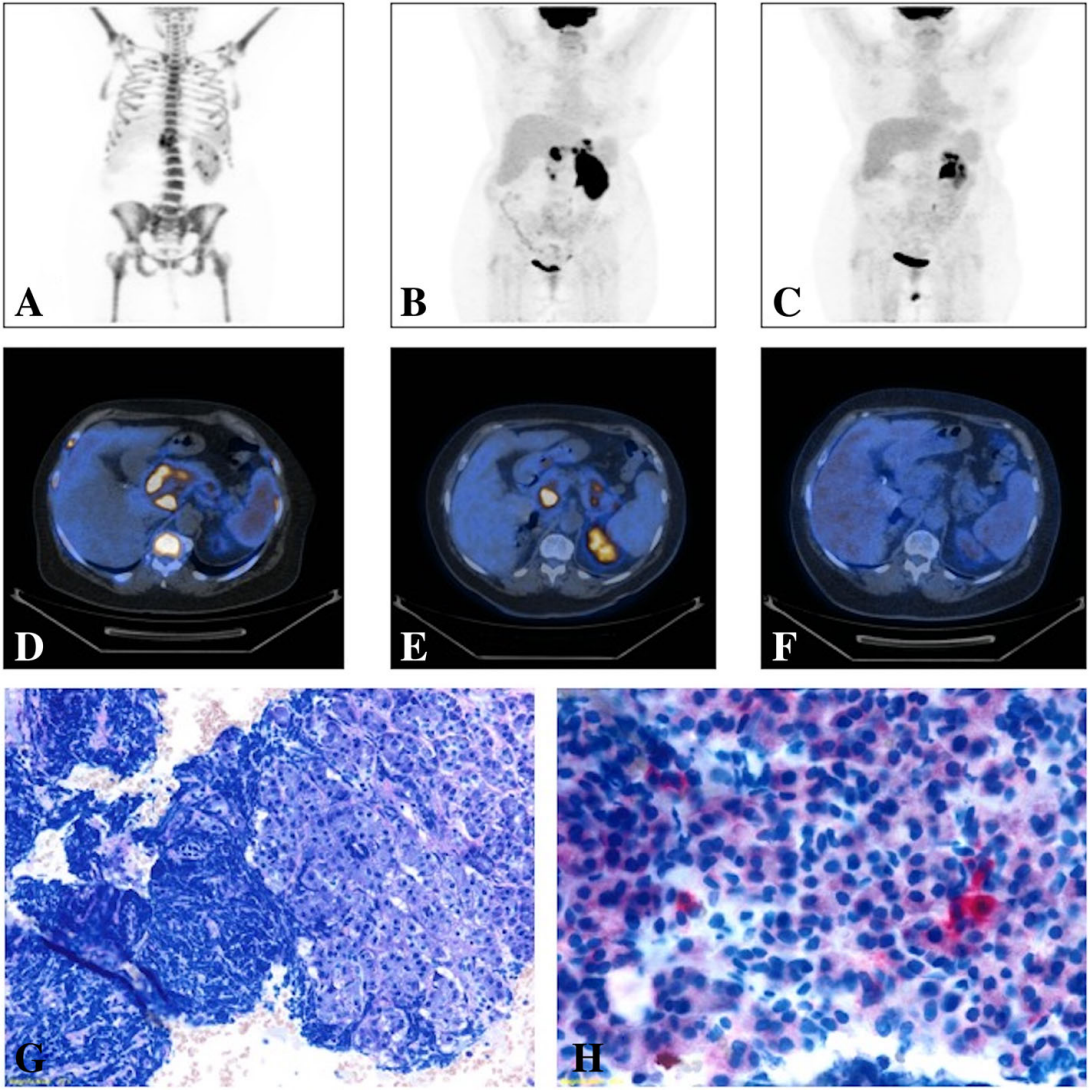
Imaging and Histological analysis from Case 1 patient: (**a**) MIP image (PET-CT scan) before the beginning of IO treatment showing multiple areas of pathological hypermetabolism at patient’s skeleton, spleen and pancreas (SUV max = 32); (**b**) MIP image (PET-CT scan) after the first course of IO showing the disappearance of the diffuse uptake at patient’s skeleton, a slight reduction of the pancreatic lesion previously reported, with the onset of new areas of pathological uptake within pancreas, gastric fundus and antrum and left renal parenchyma (Deauville V – Progressive Disease); (**c**) MIP image (PET-CT scan) after the second and last IO course showing a complete metabolic response (Deauville I), with the disappearance of all the previously described lesions; (**d**) Axial section of a PET-CT scan performed before IO treatments showing pathologic hypermetabolism in pancreas head and body (SUV max = 32, Deauville V), spleen and skeleton (Deauville IV); (**e**) Axial section of a PET-CT scan performed after the first IO course showing a Progression of the Disease; (**f**) Axial section of a PET-CT scan performed after the second and last course of IO documenting a Complete Response. (**g**) Histological image from a Pancreatic echo-endoscopic biopsy showing pancreatic exocrine tubulo-acinar secretory units with massive infiltration represented by B-ALL neoplastic cells (Light Microscopy, Wright-Giemsa); (**h**) Histological image from a Pancreatic echo-endoscopic biopsy with specific immunostaining showing CD22 slight positivity of B-ALL blasts (Light Microscopy)

After about 1 year and a half of sustained MRD negativity, blasts were documented at the peripheral blood smear. She performed a 18F-PET/CT (PET-CT), considering the recent history of renal cancer, that documented the presence of multiple lesions (Fig. 1a and d) [5], including a large pancreatic one. In order to define the following therapeutic approach, a differential diagnosis between renal cancer metastasis and EM-ALL localization was required. A pancreatic eco-endoscopic biopsy was performed, revealing a population of CD19+ and CD22+ lymphoid cells (Fig. 1g,h). Blast cells’ CD22 positivity suggested an approach with IO, which was obtained as compassionate use. IO was administered weekly in hospitalized regimen, for a total of three infusions (1,3 mg on day 1; 0,8 mg on day 8 and 15). Therapy was well tolerated, and no adverse events occurred. As expected, the bone marrow evaluation showed a morphologic CR, even with MRD positivity (10^-3^). EM disease was still present, though, as detected by a PET-CT scan, which showed a slight reduction of the pancreatic lesion previously reported, with the onset of new hypermetabolic areas (Fig. 1b, e). Nevertheless, a second course of weekly IO was administered, for a total of four infusions (0.8 mg per dose). Surprisingly, the further PET-CT-scan documented a complete metabolic response (CMR) (Fig. 1c, f) associated with bone marrow MRD negativity. Currently, the patient is in good clinical conditions and still on IO (course 4), waiting for the identification of a matched-unrelated donor, not yet available, to proceed to allogenic bone marrow transplantation (allo-BMT).

### Case2

**Fig. 2:**
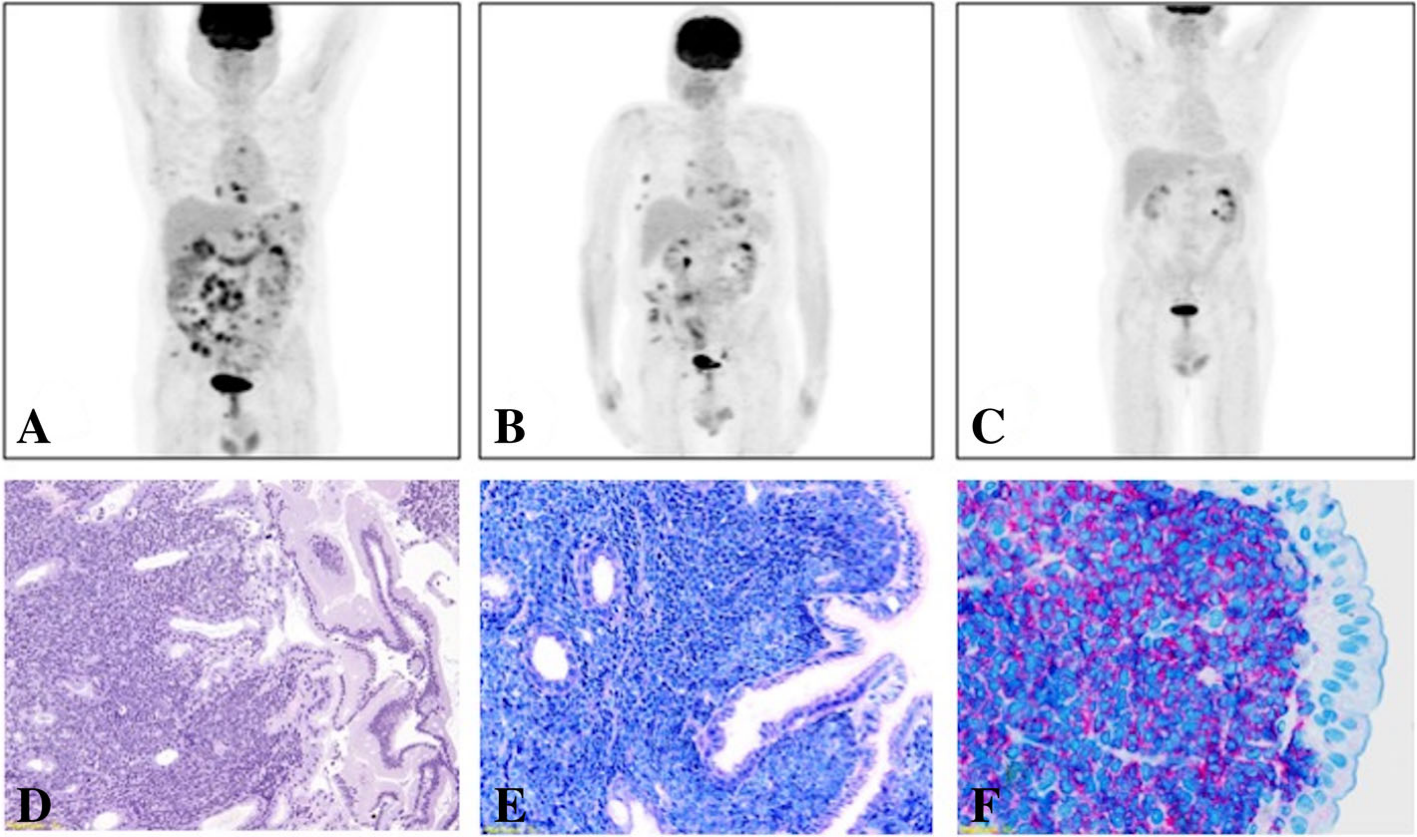
Imaging and Histological analysis from Case 2 patient: (**a**) MIP image (PET-CT scan) performed before the beginning of IO showing an multiple areas of pathological uptake localized at mediastinum, pectoral, axillary, gastric, mesenteric and iliac region (SUV max = 7.7); (**b**) MIP image (PET-CT scan) performed after the first course of IO showing a reduction of almost all the previously detected lesions, considered a Partial Metabolic Response (Deauville IV); (**c**) MIP image (PET-CT scan) performed after the second and last IO course showing the resolution of all the previously described lesions, with the persistence of slight pathological uptake next to the gastric great curvature and the hepatic hilus (SUV max = 4, Deauville IV); (**d**) Histological image from a duodenal biopsy documenting the epithelial surface of intestinal villa and submucosa diffusely infiltrated by blasts cell (Light Microscopy, H&E); (**e**) Histological image from a Duodenal biopsy showing the duodenal surface with the brush border on the epithelium and the submucosal populations of B-ALL neoplastic cells (Light Microscopy, Wright-Giemsa); (**f**) Histological image from a Duodenal biopsy with specific immunostaining showing CD22-positive B-ALL blasts (Light Microscopy)

A 67 years old man, suddenly presented muco-cutaneous bleedings associated with severe thrombocytopenia (PLT 13.000/μL), mild anemia (Hb 8.3 g/dL) and marked hyperleukocytosis (WBC 63.000/μL, 93% blast cells). The bone marrow examination showed a remarkable lymphoblast infiltration (TdT+, CD79a+, CD22+, CD19). Conventional cytogenetic analysis revealed t(9;22). Therefore, the diagnosis of BCR-ABL1 (p190)-positive B-ALL was made. The patient received a 7-day steroid pre-phase followed by ponatinib (Iclusig) at the initial dose of 15 mg daily, rapidly increased to standard dosage, 45 mg/daily, associated with monthly medicated lumbar punctures (methotrexate, cytarabine and dexamethasone), according to the GIMEMA LAL1811 clinical trial (NCT01641107). He obtained a morphological CR after 10 days of ponatinib, while MRD evaluated by real-time PCR never reached values below 0.003 copies (assessed by BCR-ABL/ABL ratio). Therapy was well tolerated and continued for 18 months, until the patient presented a painless skin lesion on the forehead, that turned out to be a CD19+ CD22+ EM-ALL localization. Bone marrow was still negative for leukemic infiltration. After local radiotherapy (4000 cGy) the skin lesion completely resolved, but the PET-CT scan control, performed just 2 months later, revealed multiple new hypermetabolic thoracic and abdominal lesions. Therefore, the patient received two courses of chemotherapy, according to BFM schedule. Therapy was complicated by an episode of gastrointestinal bleeding (melena with severe anemia) caused by duodenal and gastric disease localizations, documented bioptically (Fig. 2d-f). The PET-CT scan after the chemotherapy courses (Fig. 2a) showed a remarkable disease progression (PD). Immunohistochemistry of duodenal biopsy had showed CD22 positivity, suggesting a possible efficacy of IO therapy, obtained as compassionate use. He underwent two IO courses according to standard schedule without the occurrence of any adverse event. The following bone marrow analysis documented a persistent morphological and cytogenetic CR, but still MRD positivity (BCR-ABL/ABL 0.03 copies). The PET-CT scan showed the progressive decrease of all previously described lesions (Fig. 2b, c), reaching a partial metabolic response (PMR), because of the persistence of a pathologic hypermethabolic areola. The patient proceeded to allo-BMT 1 month later. No GVHD occurred, and no VOD/SOS (Veno-occlusive disease/Sinusoidal occlusive syndrome) was observed. To sum up, the patient had several EM “outbreak” relapses, while the bone marrow morphologic complete remission with MRD positivity was maintained. None of the therapies he underwent (TKI, local radiotherapy, chemotherapy, IO) lead to MRD negativity. However, notably, only IO induced an important reduction of extramedullary disease localizations.

## Discussion and conclusion

Thanks to its good safety profile and a wide experience in clinical practice, blinatumomab is currently considered the first therapeutic choice in R/R Ph- ALL [6, 7]. However, there are at least two still-open issues about its effectiveness: the occurrence of CD19- immune escape, and its poor effect on EM disease. Firstly, loss of CD19 has been demonstrated to represent a key element of resistance [3]. Splice variants, point mutations, lineage switch, CD81 deficiency are some of the better described CD19- relapse mechanisms [8–11]. Moreover, as recently reported, a high count of T reg lymphocytes, and an impaired T reg/ T eff ratio, have been described to be predictive for blinatumomab resistance, mainly due to interleukin-10 production, resulting in suppression of T-cell proliferation and reduced CD8-mediated lysis of ALL cells [12]. Secondly, to the best of our knowledge, studies investigating or explaining the low efficacy on EM disease are still lacking.

Furthermore, Aldoss et al. suggested that a previous history of EM disease localization before blinatumomab administration predicts a lower CR rate (*p* < 0.05), EM relapse, and progression during therapy. One of the most interesting findings was that, in about 20% (5/32) of non-responders, a marrow CR with evidence of simultaneous leukemia progression in EM sites was documented [3].

On the other hand, IO has shown similar CR and Overall Survival (OS) rates with a different safety profile [13, 14]. A phase III randomized study (INO-VATE) demonstrated the superiority of IO compared to standard chemotherapy in terms of CR rates, underlining the potential role of the drug as a bridge-to-transplant option [15]. IO consists of a toxin (calicheamicin) conjugated to an anti-CD22 antibody [2], which allows its delivery to blast cells; hence, its toxicity does not seem to involve immune cells and, differently from what reported concerning blinatumomab, no cases of CD22 antigen loss have been described so far. Moreover, lineage switch mechanism (myeloid conversion), described mostly in CAR-T cell therapy, does not seem to involve CD22 expression: the antigen is maintained on the intermediate phenotype relapses, suggesting that simultaneous pressure on CD19 and CD22 might avoid this resistance mechanism [13]. Although experimental studies have not specifically evaluated the effectiveness of IO on EM disease, its mechanism of action suggests that EM-lesion’s microenvironment and resident cell subpopulations may have a more negative influence on blinatumomab than on IO effectiveness.

These case reports, coming from our single-center experience (totally 17 patients treated with IO) described the use of IO in EM-ALL relapses in two different settings. In both cases IO was the therapeutic choice, based on CD22 positivity assessed by immunohistochemistry on EM-lesion biopsy (Figs. 1g, h, 2d-f). Both patients achieved a bone marrow CR (Case 1 MRD negativity too) and a significant resolution of the EM involvement (1 CMR and 1 PMR), allowing them to be eligible for allo-BMT.

In conclusion, despite the mild effect of immunotherapy (blinatumomab and CAR-T) on EM disease localizations has been widely described in cancer, we hypothesize that IO could represent a valid and promising therapeutic choice in this particular and dismal setting. Moreover, we suggest the assessment of CD22 status at diagnosis and relapse in all B-ALL patients, in order to better evaluate the indication for IO use. Further studies are strongly needed to confirm our hypothesis.

## Abbreviations

Allo-BMT: Allogenic stem cells Bone Marrow Transplantation
B-ALL: B origin Acute Lymphoblastic Leukemia
CMR: Complete Metabolic Response
CNS: Central Nervous System
CR: Complete Remission
EM-ALL: Extra Medullary Acute Lymphoblastic Leukemia
H&E: Hematoxylin and Eosin
Hb: Hemoglobin
IO: Inotuzumab ozogamicin
MIP: Max Intensity Projection
MRD: Minimal Residual Disease
PD: Progression of Disease
PET-CT: 18F-PET/CT
PLT: Platelets
PMR: Partial Metabolic Response
R/R: Relapsed / Refractory
SUV: Standardized Uptake Value
TKI: Tirosine Kinase Inhibitor
VOD/SOS: Veno-occlusive disease/Sinusoidal Occlusive Syndrome
WBC: White Blood Cells
